# Secondary Structure Prediction of Protein Constructs Using Random Incremental Truncation and Vacuum-Ultraviolet CD Spectroscopy

**DOI:** 10.1371/journal.pone.0156238

**Published:** 2016-06-07

**Authors:** Mária Pukáncsik, Ágnes Orbán, Kinga Nagy, Koichi Matsuo, Kunihiko Gekko, Damien Maurin, Darren Hart, István Kézsmárki, Beata G. Vertessy

**Affiliations:** 1 Institute of Enzymology, Research Centre for Natural Sciences, Hungarian Academy of Sciences, Budapest, Hungary; 2 Department of Physics, Budapest University of Technology and Economics and MTA-BME Lendület Magneto-optical Spectroscopy Research Group, 1111 Budapest, Hungary; 3 Hiroshima Synchrotron Radiation Center, Hiroshima University, Higashi-Hiroshima, Japan; 4 Institut de Biologie Structurale (IBS), CEA, CNRS, University Grenoble Alpes, Grenoble 38044, France; 5 Department of Applied Biotechnology, Budapest University of Technology and Economics, Budapest, Hungary; Universitetet i Bergen, NORWAY

## Abstract

A novel uracil-DNA degrading protein factor (termed UDE) was identified in *Drosophila melanogaster* with no significant structural and functional homology to other uracil-DNA binding or processing factors. Determination of the 3D structure of UDE is excepted to provide key information on the description of the molecular mechanism of action of UDE catalysis, as well as in general uracil-recognition and nuclease action. Towards this long-term aim, the random library ESPRIT technology was applied to the novel protein UDE to overcome problems in identifying soluble expressing constructs given the absence of precise information on domain content and arrangement. Nine constructs of UDE were chosen to decipher structural and functional relationships. Vacuum ultraviolet circular dichroism (VUVCD) spectroscopy was performed to define the secondary structure content and location within UDE and its truncated variants. The quantitative analysis demonstrated exclusive α-helical content for the full-length protein, which is preserved in the truncated constructs. Arrangement of α-helical bundles within the truncated protein segments suggested new domain boundaries which differ from the conserved motifs determined by sequence-based alignment of UDE homologues. Here we demonstrate that the combination of ESPRIT and VUVCD spectroscopy provides a new structural description of UDE and confirms that the truncated constructs are useful for further detailed functional studies.

## Introduction

Detailed knowledge of protein three-dimensional structure is indispensable for understanding the mechanism of protein action. As of present, macromolecular X-ray crystallography and multidimensional NMR are the techniques of choice. Despite numerous advances in both these methodologies during recent years, several limitations still exist. For a detailed 3D structural determination by multidimensional NMR, the size of the protein is an important factor, and proteins larger than 30 kDa pose serious difficulties preventing structural determination [1]. There is no such size limitation in macromolecular X-ray crystallography, in this case, however, the need for well-diffracting crystal specimens is still a major bottleneck, especially in unstructured or flexible sequences that lack clearly identified domains. Methods to predict and help the design of crystallizable protein constructs are therefore highly required. Generation and investigation of such deletion constructs may then be a first and important step towards characterization of full-length proteins.

In the case of multidomain proteins, the easiest way to create shorter constructs is truncation via PCR cloning. In contrast to PCR-based methods that rely on rational design of primers (implying some knowledge of domain structure *a priori*), the random screening method ESPRIT **(e**xpression of **s**oluble **p**rotein by **r**andom **i**ncremental **t**runcation) uses an exonuclease III based protocol [2,3] to generate a library of expression constructs containing all possible gene deletions. Thus every point in the gene is tested as a start and/or stop position. Large deletions may result in isolated domains, whilst short deletions have been shown to rescue soluble expression of nearly full-length proteins [4,5]. In some cases, multiple soluble constructs are identified by screening it then becomes necessary to select some of these numerous constructs prior to detailed structural studies. Probably the most important aspect guding these selections is the degree of foldedness of the soluble fragments and several biophysical methods can be used to this end; the results may then identify the most promising candidates.

In contrast to the above mentioned methods of X-ray crystallography and NMR for the reconstruction of 3D protein structure with atomic resolution, circular dichroism (CD) spectroscopy is generally thought to provide only limited structural information via the excitation of peptide bonds by ultraviolet light. CD is mostly used to measure the ratio of the different secondary structure components in proteins [6–9]. However, recent developments of CD spectroscopy to exploit the infrared and X-ray photon-energy regions have opened new avenues through probing molecular vibrations [10] and core electron excitations [11], respectively; this makes possible a spatially specific structural analysis. Still, determination of the full secondary or tertiary structure of proteins requires the analysis of the CD spectra using *ab initio* or neural network calculations based on their amino acid sequence [12].

The aim of this study was to determine if a combination of vacuum ultraviolet circular dichroism (VUVCD) spectroscopy with ESPRIT, a state-of-the-art library expression method, would permit experimental mapping of the full secondary structure of a protein without prior knowledge, other than that of the amino acid sequence. We performed the study on the uracil-DNA degrading factor (UDE) of *Drosophila melanogaster*, which recognizes and removes uracil from DNA at the end of the third larval stage [13]. The fruit fly genome lacks the otherwise common uracil-DNA glycosylase and can tolerate uracil incorporation in its DNA [14]. We showed that UDE has close homologues only in the genomes of other pupating insects: no function has yet been attributed to any of these homologues. UDE does not show any sequence similarity to known uracil-processing and -excising glycosylases or nucleases [13]. Therefore, characterization of the structure of this novel DNA-degrading protein is expected to provide significant novel insights into uracil-DNA recognition. Limited proteolysis showed extensive protection by DNA along duplicated conserved sequence Motifs 1A and 1B, and *de novo* modelling of Motif 1A and 1B predicted similar α-helical bundles and two conserved positively charged surface patches for both motifs. These results suggested that DNA binding may occur at the N-terminal segment of UDE that contains Motifs 1A and 1B [15]. Despite numerous crystallization efforts, no protein crystals were obtained, perhaps due to the predicted very high degree of conformational freedom of several protein segments [16]. UDE therefore presented an interesting object for the present study.

In the present work, we generated tens of thousands of randomly truncated UDE constructs. Following selection of best soluble hits, we performed thermostability and gel filtration studies to evaluate the folded nature of the constructs. We then analyzed the secondary structural organization of these constructs in a side-by-side comparison with full-length UDE using VUVCD spectroscopy. Quantitative analysis of CD data using the SELCON3 program and neural network enabled us to designate potentially new domain boundaries.

## Materials and Methods

### Cloning of UDE inserts into pESPRIT002 vector

Three starting constructs were generated by amplifying the *ude* gene (GenBank accession no. CG18410) with one common forward primer (UDEfor1 5’- gatcc taggg cgcgc cgatg attaa gtgcc atatg ccgtc gagtt ggaga cggc-3’) and three reverse primers (UDErev1 5’-gatcc tagat gcatt ctccc tcttc ttctt ccttt tgggc-3’, UDErev2 5’-gatcc tagat gcatt cagct tttcg atgta ctgct tcagc-3’, UDErev3 5’-gatcc tagat gcatt gagga aatcc tcaag tacct ggact cc-3’); the reverse primers encoded 3 separate C termini (Leu-210, Leu-330, Glu-370). PCR products were cloned into pESPRIT002, a pET9a derivative resulting in direct fusions to a downstream biotin acceptor peptide [17] used for solubility detection, and with upstream *Asc*I and *Aat*II restriction sites for construction of 5’ DNA deletion (N-terminal truncation) libraries. Similarly, another three starting constructs were generated by amplifying the *ude* gene with one reverse primer (UDErev4 5’- gatcc taggc ggccg ctcac tcctc cctct tcttc ttcct tttg-3’) and three forward primers (UDEfor2 5’-gatcc tagga cgtcg atgat taagt gccat atgcc gtcga gttgg agacg-3’, UDEfor3 5’-gatcc tagga cgtcg tggag acggc tacgc aaaat cagt-3’, UDEfor4: 5’-gatcc tagga cgtcg aatgg cggag gggcg tccag c-3’) encoding fixed N-termini (Met-1, Trp-25, Asn-126). These PCR products were cloned into pESPRIT002 as *Aat*II and *Not*I fragments in-frame with the hexahistidine tag and TEV sequence, and permitting construction of 3’ DNA deletion (C-terminal truncation) libraries via downstream *Not*I and *Nsi*I sites.

### UDE truncation library synthesis

The procedures were described previously [3,18]. Briefly, high quality, unnicked plasmid was prepared from 200 ml overnight culture using a classic protocol for alkaline lysis, phenol chloroform extraction and isopropanol precipitation. Residual salts and RNA were removed using a Qiagen Minprep Kit to repurify the initial plasmid preparation. For 5' deletion libraries, 10 μg of an equimolar mix of the three pESPRIT002 plasmids were digested with *Aat*II and *Asc*I and, similarly, for 3' deletion, 10 μg of the other three pooled plasmids were digested with *Nsi*I and *Not*I; these enzyme pairs yield an exonuclease III insensitive 3' overhang and an exonuclease sensitive 5' overhang. Next, 4 μg linearised plasmid DNA (final conc. 33.3 ng/μl) were incubated in NEB buffer 1, 70 mM NaCl and 400 U of Exonuclease III (at 100 U/μl) in a final volume of 120 μl at 22°C. To ensure even fragment distribution, every 60 s, 1/60th reaction volume (2 ul) was transfered to a quenching tube (200 μl of 3 M NaCl) on ice; this was then heated to 70°C for 20 min and purified using a Machery-Nagel Nucleospin Extract II kit according to the kit protocol. In order to remove the long 5′ overhang left following exonuclease III digestion, the vectors were treated with 5 U of Mung Bean Nuclease in 1× Mung Bean buffer at 30°C for 30 min, then purified with the Nucleospin Extract II kit. The ends of the linearised plasmids were polished by incubation with 5U *Pfu* polymerase in 1× *Pfu* polymerase native buffer, 2.5 mM dNTPs) at 72°C for 20 min. For size fractionation of DNA inserts, the truncated linearised plasmids were electrophoresed in a 0.5% agarose gel each library excised as 0–50% and 50%-100% *ude* gene lengths (thereby resulting in small and large sub-libraries for each library). Plasmids were extracted from agarose using QIAexII kit (Qiagen) and recircularised with T4 DNA ligase, then used to transform OMNImax *E*. *coli* cells (Life Technologies). Transformation mixes were recovered in SOC medium and titrated on LB agar Petri dishes supplemented with kanamycin 50 μg/ml. Remainder of SOC mix was placed at 4°C.

### Colony blot analysis

*E*. *coli* BL21 (DE3) competent cells were transformed with the four UDE sub-libraries, plated on 22 cm LB agar QTrays (kanamycin 50 μg/ml and chloramphenicol 30 μg/ml) and incubated overnight at 37°C. From both 5’ and 3’ deletion libraries, approximately 28 000 colonies were picked robotically into 384 well plates containing 80 μl TB medium per well (kanamycin 50 μg/ml and chloramphenicol 30 μg/ml). Liquid cultures were grown overnight in a HiGro incubator (Digilab) at 37°C, 300 rpm. Clones were arrayed robotically onto a nitrocellulose membrane laid over 22 cm LB agar plates (kanamycin 50 μg/ml and chloramphenicol 30 μg/ml) and incubated overnight at 25°C until colonies on the membrane were just visible. Next day the membrane was moved onto a fresh pre-warmed LB agar plate (supplemented with antibiotics, arabinose 0.2% w/v and 50 μM biotin) to induce protein expression at 30°C for 4–5 h. Membranes were then placed on filter paper soaked in denaturing buffer (0.5 M NaOH, 1.5 M NaCl) and incubated 10 min at room temperature to lyse colonies as described [19]. The membranes were neutralised for 5 min in neutralization buffer (1 M Tris, 1.5 M NaCl, pH 7.5) and immersed in 2×SSC buffer for 15 min. The remaining colony debris on the membranes were removed with a glass spreader. The membranes were blocked overnight in Superblock (Pierce) at 4°C. The membranes were washed with PBS-Tween (PBS with 0.1% Tween 20) buffer for 3 × 15 minutes and incubated in 50 ml PBS-Tween containing 16 μl anti-hexahistidine antibody for 1 h at 4°C. After washing steps with PBS-Tween buffer, the membranes were incubated in 50 ml of PBS-Tween containing 10 μl streptavidin Alexa Fluor 488 and 50 μl Alexa Fluor 532 rabbit anti-mouse IgG for 1 h at 4°C. After 3 × 15 min washing with PBS-Tween buffer and 5 min with destilled water, the membranes were scanned with a Typhoon 9400 fluorescence scanner (GE Healthcare) for hexahistidine tag and biotin acceptor peptide signal intensities respectively. Signals were quantified from digitised images using Visual Grid software (GPC Biotech) and data exported to Microsoft Excel for analysis.

### High-throughput protein expression and purification

The most fluorescent 96 clones from the UDE colony arrays were selected for small-scale protein expression in 4 ml TB (kanamycin 50 μg/ml and chloramphenicol 30 μg/ml) cultures in 24 well plates. Protein expression was induced at OD600 = 0.7 by addition of arabinose 0.2% w/v and 50 μM biotin followed by overnight shaking at 25°C. Cells were pelleted by centrifugation and resuspended in 4 ml of spheroplast buffer (20 mM Tris, 250 mM NaCl, 20% w/v sucrose and 1 mg/ml lysozyme, pH 8.0). The sphaeroplasts were centrifuged at 3700 rpm 10 min at 4°C and then resuspended in 800 μl lysis buffer (10 mM Tris, 0.5% Brij, 0.25 U/μl Benzonase, pH 7.5 and 0.8 μl Complete Protease Inhibitor Cocktail (Roche)). Protein samples were loaded onto a 96 well filter plate supplemented with 50 μl Ni^2+^NTA resin in each well and mixed by rotation at 4°C for 30 min. The samples were washed with 50 mM sodium phosphate buffer, pH 7, 300 mM NaCl, 5 mM imidazole and eluted with 50 mM sodium phosphate buffer, pH 7, 300 mM NaCl, 300 mM imidazole. The purified 96 samples were visualised by SDS–PAGE and the sequence boundaries of clones exhibiting visible purified proteins determined by DNA sequencing.

### Scale-up expression and purification of selected clones

From the 72 positive clones 9 clones were scaled up using the *BL21(DE3)ung-151pLysS E*. *coli* expression system in 500 ml LB at 37°C. Protein expression was induced at OD600 = 0.7 with 0.1 mM IPTG at 25°C for 4–5 h. The recombinant hexahistidine-tagged UDE proteins were purified on a HisTrap column in an AKTA Purifier System, with Unicorn software. Absorbance of eluates was monitored at both 260 and 280 nm. Total cell lysate was prepared from 500 ml of culture in 25 ml of lysis buffer (50 mM Tris-HCl, 150 mM KCl, and 1 mM EDTA, pH 8.0, complemented with 1 mm DTT, 0.1 mm phenylmethanesulfonyl fluoride and proteinase inhibitor cocktail). After loading, the HisTrap column was washed with buffer A (50 mM Tris-HCl, 30 mM KCl, 1 mM EDTA, and 5 mM imidazole, pH 7.5, complemented with 0.1 mm phenylmethanesulfonyl fluoride and 1 mm DTT) until and then with buffer B (buffer A also containing 1 M KCl) to elute the contaminating proteins. UDE constructs were eluted using buffer A complemented with different concentrations of imidazole (25 mM, 50 mM, 300 mM, cf S1 Fig). Protein fractions were dialyzed against 25 mM Tris-HCl, 150 mM KCl, and 1 mM EDTA (pH 7.5) and concentrated to 20–30 mg/ml in a Vivaspin centrifugal concentrator. Protein concentrations were determined by UV absorbance measurements at 280 nm using NanoDrop spectrophotometer. Extinction coefficients and molecular masses of the respective UDE constructs were calculated for the His-tagged sequences using ProtParam tool of the Expasy server. Additionally, protein concentrations were also measured by Bradford assay [20]. The difference between results provided by these two methods was less than 5%.

### Analytical gel filtration

Gel filtration was conducted on Superdex 200HR size exclusion column using an AKTA Purifier System. Absorbance of eluates was monitored at both 260 and 280 nm. Size exclusion chromatography was performed as described previously [15].

### Thermofluor

A thermofluor assay was used to measure the thermal stability of purified UDE truncated constructs. The reaction mix contained 0.25 mg/ml or 0.5 mg/ml or 1 mg/ml of purified UDE fragments, 1 μl of 40 × diluted Sypro Orange dye, and 25 mM Tris-HCl, 150 mM KCl, and 1 mM EDTA (pH 7.5) in 25 μl final volume. The mix was transferred to a 96 well plate and analysed with a Stratagene Mx3000P real-time PCR Thermal Cycler by increasing the temperature from 25°C to 99°C at a rate of 0.5°C /min [21].

### Vacuum ultraviolet circular dichroism (VUVCD) spectroscopy

Investigation of the secondary structure of UDE by means of VUVCD spectroscopy has been performed at beamline #15 of Hiroshima Synchrotron Radiation Center (HiSOR) using a high photon flux of 1010 photon/s. Circular dichroism spectra were measured using a polarization modulation technique over the wavelength range of 170–255 nm with a resolution of 1 nm [9]. Details of the setup are reported elsewhere [22]. Due to rapidly increasing water absorption for wavelength shorter than 180 nm, we kept the light path within the samples as short as ~12 μm and used solutions in 10 mM NaPi (pH = 7.5) buffer with the protein concentrations 6 mg/ml or 9 mg/ml and 3 mg/ml in the case of CA7.

The details of the optical end electronic setup of the VUVCD spectrophotometer are described in previous papers [22–24]. The calibration of the amplitude of ellipticity and wavelength position was confirmed by monitoring the CD spectrum of a reference sample [25,26], in our case the aqueous solution of ammonium d-camphor-10-sulfonate.

The optical cell used in our measurements is a demountable cell for which the technical details have been described in [26]. There is no sample evaporation during the measurement, even if the sample is held under high vacuum for 10 hours [22]. The accuracy and reproducibility of the absolute pathlength was tested using standard calibration materials (ACS and whale myoglobin) [26]. The reproducibility of the spectra after disassembling and refilling the sample cell is within less than 5%. The spectra were recorded with a 16-s time constant, 4 nm/min scan speed, and the final spectra were obtained by averaging 8–10 accumulations and subtracting the signals measured on the buffer solution. The temperature of the cell was kept at 293 Kelvin degree.

**Fig 1:**
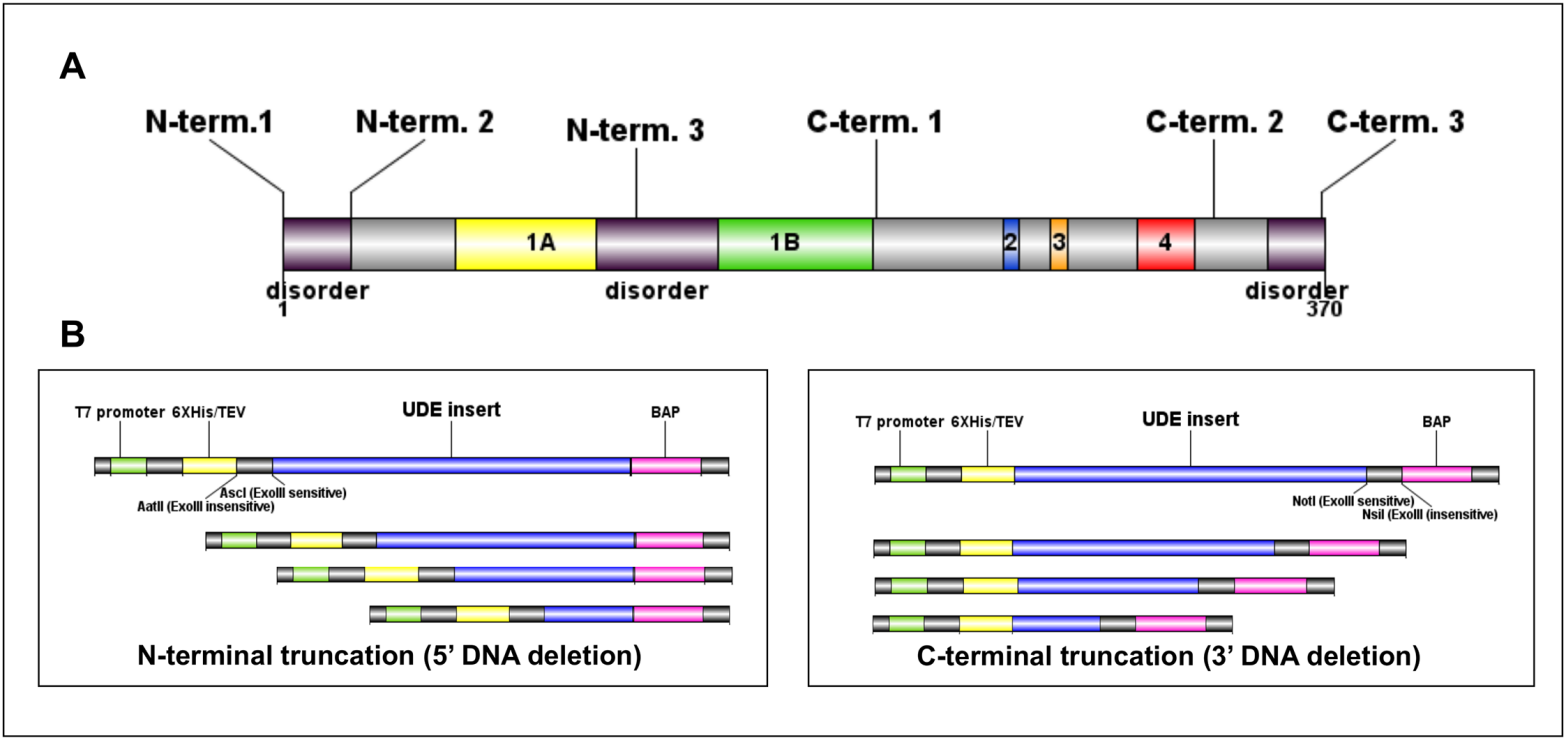
Unidirectional truncation of UDE gene. **(A)** The location of designed starting points along the UDE sequence indicating the predicted disordered segments and the conserved motifs. **(B)** The truncated UDE gene fragments generated by N-terminal truncation were fused in-frame with the biotin acceptor peptide and out-of-frame with hexahistidine tag, while fragments produced by C-terminal truncation were fused in-frame with hexahistidine tag and out-of-frame with BAP.

Fluctuations in photon flux during measurements of individual spectra as well as the long-term variation of light intensity are eliminated by the optical servo-control system realized at the BL15 beamline at the Hiroshima Synchrotron Radiation Center (cf Fig 1 and the corresponding main text in [22]).

The CD spectrum of a given sample was obtained by using the aforementioned calibration procedure. For each fragment, the CD spectrum is the result of 8–10 consecutive measurements from which the baseline spectrum of the buffer is subtracted. Delta epsilon spectrum of a given protein/fragment was obtained by dividing the measured CD spectrum by the protein concentration and the optical path length; and by multiplying it by the mean residue weight. The latter quantity was determined using the total molecular weight of the fragment and the number of amino acids in the sequence.

### Secondary structure analysis

The secondary structure components and the number of segments of full-length UDE and UDE constructs were determined using the SELCON3 program and the VUVCD spectra of 31 reference proteins with known X-ray structures [27–30]. The sequence based prediction of the positions of the secondary structure components along the amino acid sequences of UDE and UDE constructs were carried out by combining VUVCD analysis with the NN algorithm. The VUVCD-NN method was detailed previously [31].

## Results

### Construction of UDE expression libraries by ESPRIT

**Fig 2:**
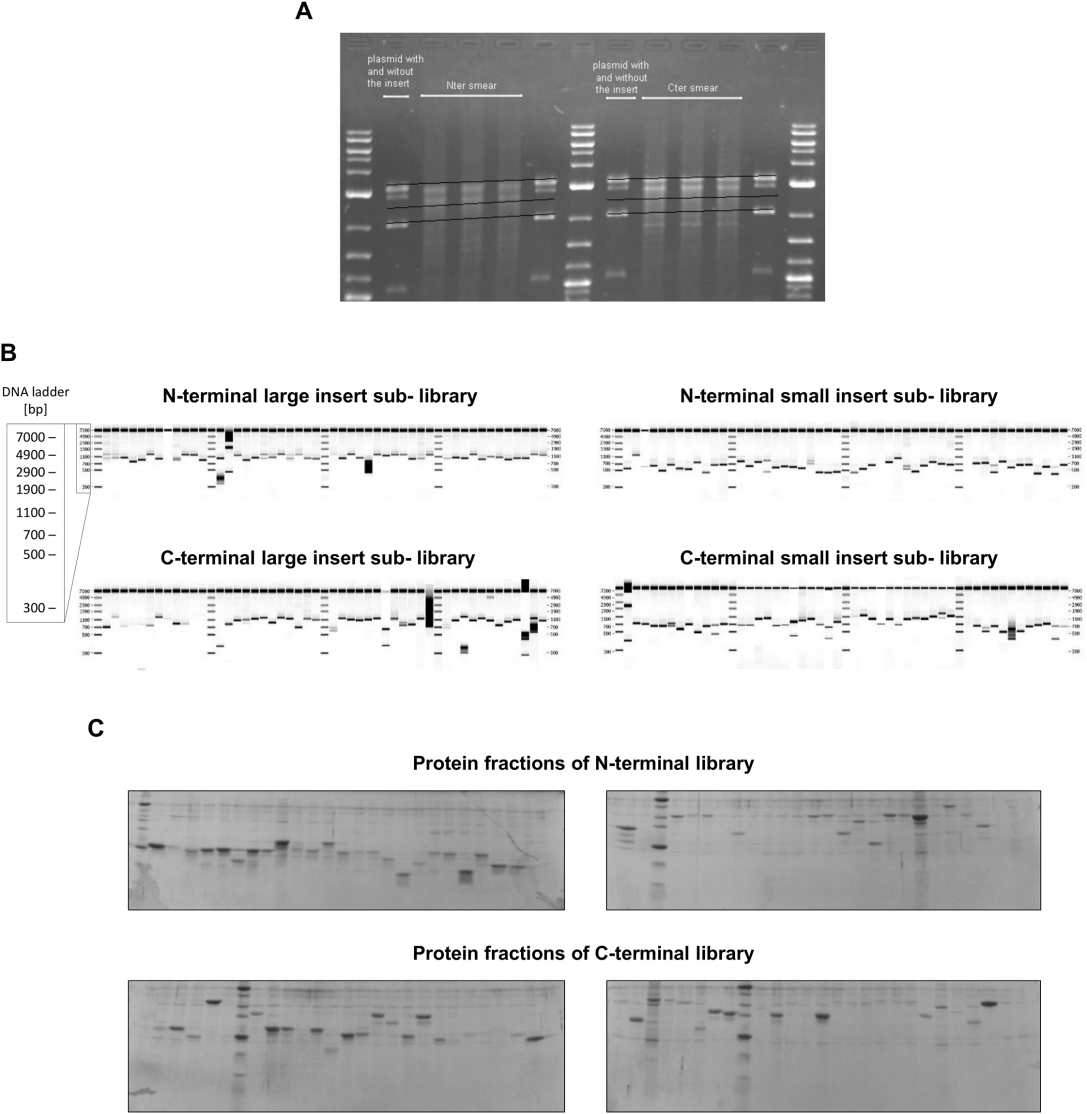
Screening expression level and solubility of UDE truncation libraries. **(A)** Size fractionation of UDE fragments generated by unidirectional truncation on agarose gel. In the lanes next to the DNA ladders is the vector with total length UDE gene at higher position while the empty vector is at a lower position. N1–N3 and C1–C3 marked samples show by the exonuclease III truncation generated UDE constructs. **(B)** Assessment of UDE sublibraries size and diversity by PCR screen. **(C)** Separation of purified protein fractions on Ni^2+^-NTA resin from N-terminal (upper panels) and C-terminal (bottom panels) libraries on SDS-PAGE.

Limited proteolysis data strongly suggested that UDE possessed a multidomain structure. Since UDE has few homologues for multiple sequence alignment construction, we have been unable to accurately define these domain boundaries using bioinformatics. Expression of soluble proteins using a library approach based upon random incremental truncation (ESPRIT) was therefore used to create a comprehensive oversampled unidirectional truncation library from a full-length *ude* gene [2–5,13] from which soluble variants were identified by screening. Sequence-based structural disorder predictions by RONN, IUPred and DISOPRED suggested flexible segments at the N- and C-terminal ends of the protein, as well as in the linker region between Motifs 1A and 1B [15]. Based on these data we designed six starting constructs to be unidirectionally truncated from either the 5’ or 3’ end of the *ude* gene (Fig 1). The six UDE inserts were cloned into the plasmid vector pESPRIT002 (a pET9a-derived vector) that introduces restriction sites with exonuclease III sensitive and resistant overhangs at the termini to be truncated [32] and encoding an N-terminal hexahistidine tag and a C-terminal biotin acceptor peptide (BAP) [17] used here as an indicator of soluble expression (Fig 1). Controlled exonuclease III and mung bean nuclease digestion, followed by vector recircularisation with DNA ligase resulted in fusion of the truncated UDE fragments to the hexahistidine tag with TEV protease cleaving site in the case of the N-terminal deletions; and to the BAP in the case of the C-terminal deletions. Truncated target genes in vectors were size fractionated by excision from agarose gels resulting in four (small/large and N-terminal/C-terminal) sublibraries (Fig 2A). Size distribution was verified for each sublibrary (Fig 2B) by colony PCR of 48 colonies from each sublibrary with vector specific insert flanking primers. To summarise, each unidirectional truncation reaction (5’ or 3’) was performed on a pool of 3 starting constructs with varying fixed ends; for each unidirectionally truncated library, inserts were size selected into small and large size ranges. The four sublibraries were finally transformed to *E*. *coli* BL21 AI RIL cells for screening of expression level and solubility.

### Screening of libraries and scale-up

Approximately 28,000 colonies were robotically picked into 384-well microtiter plates. Consequently, each construct was sampled two times on average for its expression and putative solubility. Since it is impossible to directly measure solubility characteristics of thousands of constructs, the method uses a short C-terminal biotin acceptor peptide [17] as a proxy for soluble expression: if the resulting protein fragment is soluble, the endogenous BirA enzyme of *E*. *coli* mediates more efficient *in vivo* biotinylation of the target than when the construct is degraded or insoluble [19,33]. 96 hits from UDE colony blot analysis (cf Materials and Methods) were selected for small-scale protein expression. These constructs were expressed in 4 ml cultures in 24-well plates and purified on Ni^2+^ NTA resin. Eluted fractions were then analysed using SDS-PAGE to confirm solubility and purifiability (Fig 2C). In total, 72 positive clones were sent for sequencing to identify the construct boundaries yielding soluble protein fragments.

**Fig 3:**
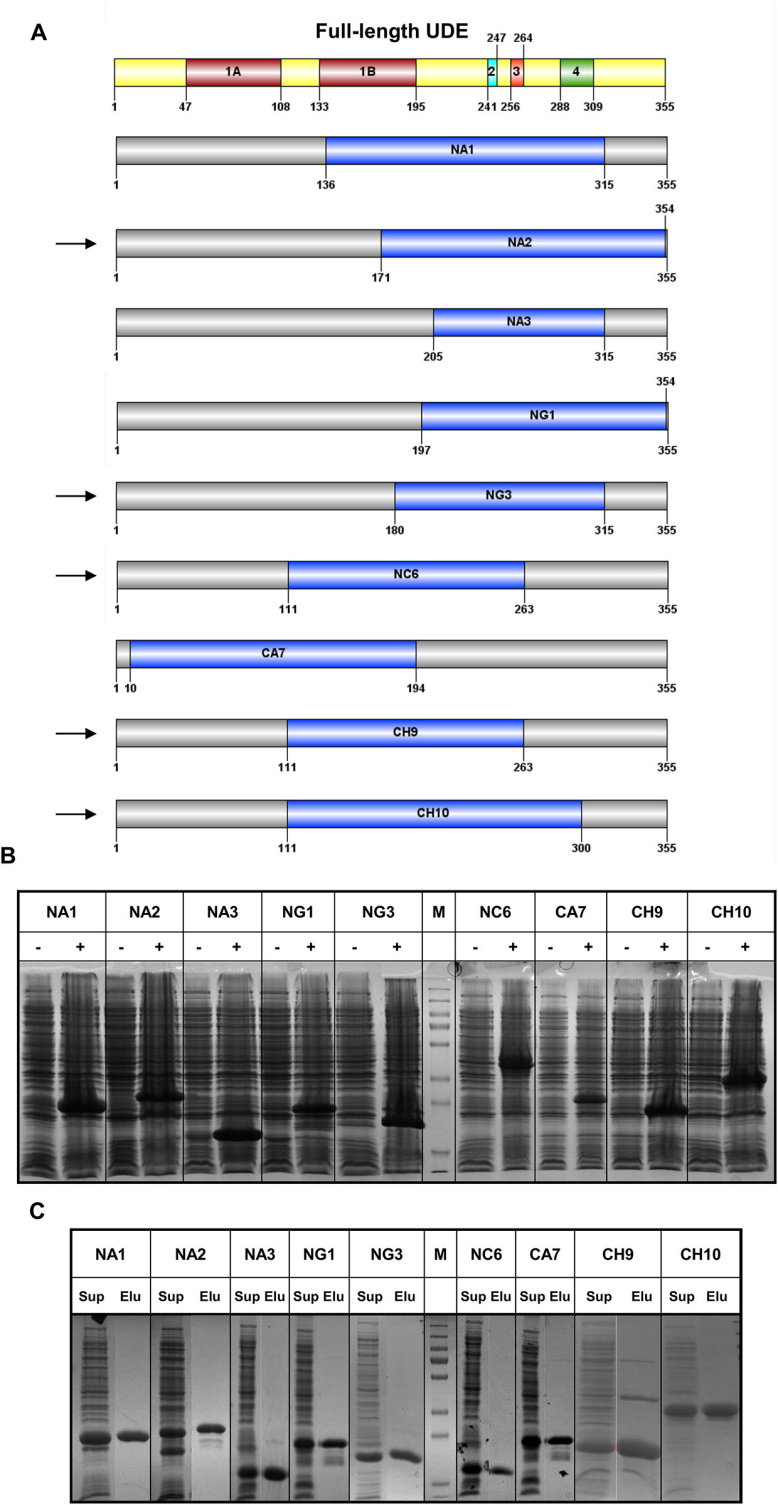
Alignment and scale-up of selected UDE truncated fragments. **(A)** The restricted nine UDE truncated fragments from the identified protein clusters that were chosen for scale-up. Arrows show the expression compatible boundaries compared to the previously designated conserved motifs determined by the alignment of UDE homologues sequences. **(B)** The optimized expression of the nine UDE constructs in *E*. *coli* BL21 cells before (-) and after (+) IPTG induction. **(C)** Purification of recombinant UDE constructs by Ni^2+^-affinity chromathography. Gel slice images show the supernatant (termed as “Sup” on the figure) of cell lysis and the 300 mM imidazole elution (termed as “Elu” on the figure) fractions for each constructs. Note that the entire purification process can be followed on S1 Fig that shows the whole SDS-PAGE gels, not only the supernatant and imidazole elution samples.

Based on the amino acid alignment of the sequenced truncated UDE clones, nine clusters of soluble constructs with similar size were identified. Compared to the previously designated conserved motifs determined by the alignment of UDE homologues sequences, we identified five new expression-compatible domain boundaries (Fig 3A). Data on the expression levels of constructs from the small scale trials, together with the coverage of UDE conserved segments, were used to select one truncated UDE fragment from each cluster for scale-up and further structural analysis. After optimization of the expression conditions, nine constructs were expressed at 500 ml scale, enabling purification of truncated protein constructs (Fig 3B and 3C and S1 Fig). To verify that the proteins were not aggregated, and to analyse their oligomer status, we used analytical gel filtration. Proteins formed dimers in solution except the NC6 fragment that was monomeric (Table 1). Construct CA7 exhibited high molecular weight soluble aggregates in the void elution. We also performed a detailed analysis of thermostability of the UDE deletion mutants using the ThermoFluor method [21]. Determination of the melting temperature of the UDE fragments gave partial information on protein folding. CA7 and CH9 constructs did not show a clear melting point suggesting these fragments may not have well-defined tertiary/quaternary structure (Table 1); this observation for CA7 accords well with the gel filtration data, and is suggestive of unfolded, flexible segments. For constructs NA1, NC6, NA3 and NG3 clear melting points were determined at 51.3, 53.7, 57.1 and 51.5°C respectively.

**Table 1:** The results of analytical gel filtration and the determination of the oligomeric status of each truncated fragments.

UDE constructs	Tm (°C)	Analytical gelfiltration			Oligomer status
		Vel (kDa)	Mapp (kDa)	Mcalc (kDa)	
**NA1**	51,3	62,4	42,1	25,7	**dimer**
**NA2**	53,9	56,6	57,0	26,3	**dimer**
**NA3**	57,1	69,6	28,9	18,0	**dimer**
**NC6**	53,7	55,7	35,6	34,7	**monomer**
**NG1**	56,4	60,0	47,7	23,3	**dimer**
**NG3**	51,5	64,6	37,5	20,9	**dimer**
**CA7**	-	44,3	-	25,4	**monomer**
**CH9**	-	58,3	52,1	22,1	**dimer**
**CH10**	-	56,0	58,8	32.1	**dimer**
**full length UDE**					**monomer**

V_el_: elution volume, M_app_: apparent molecular weight, M_calc_: calculated molecular weight based on column calibration. Data of thermostability analysis by Thermofluor. T_m_: melting point of the given protein.

### Secondary structure estimation by combining VUVCD results with neural network (NN) algorithm

For detailed secondary structural studies VUVCD spectroscopy measurements were carried out on the full-length UDE and its nine truncated fragments over the wavelength region of 170–255 nm at the Hiroshima Synchrotron Radiation Center [22]. Such extension of the conventional far-ultraviolet CD spectroscopy towards shorter wavelengths highly improves the reliability of the estimation of the protein secondary structure content [34,35].

**Fig 4:**
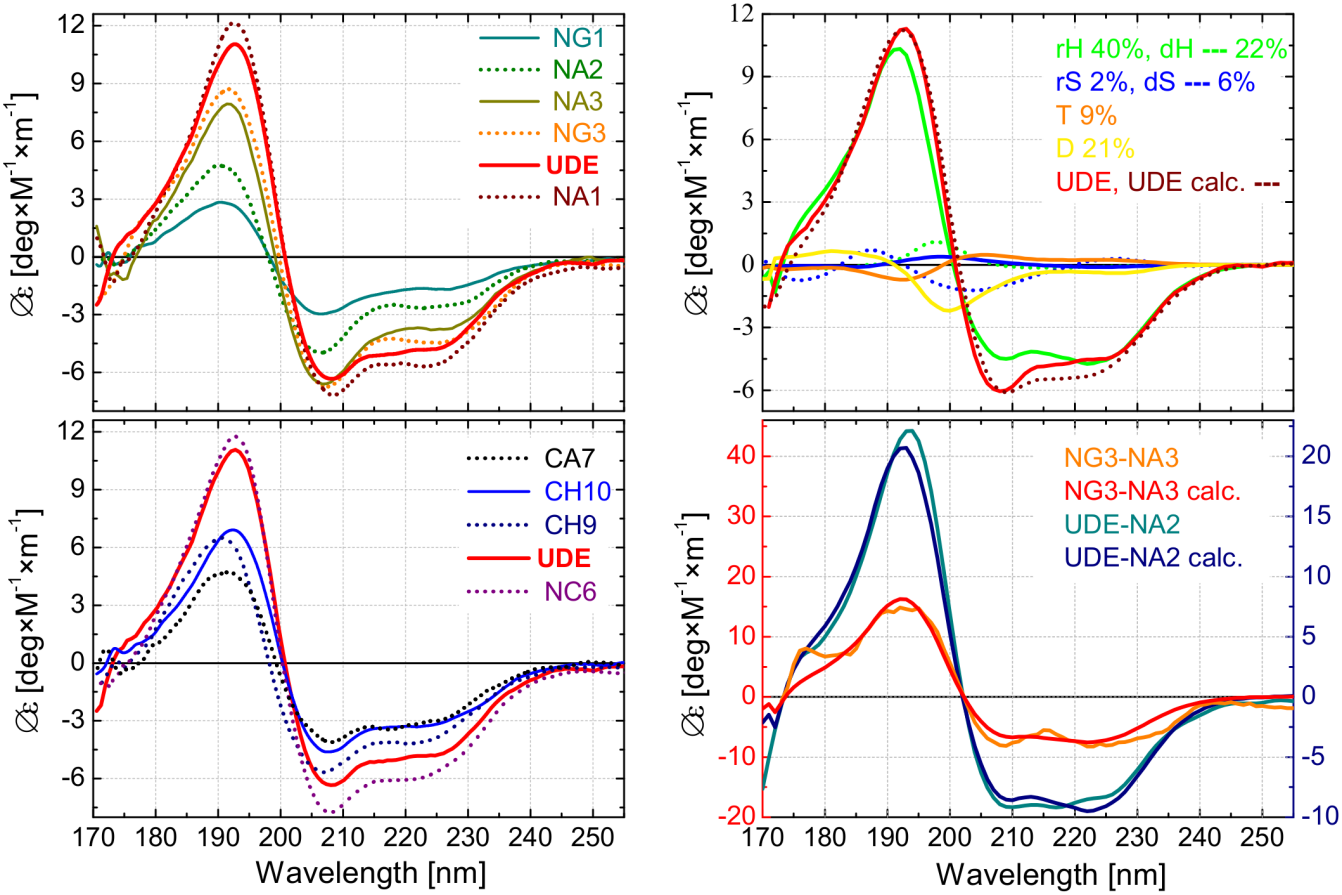
Secondary structure content determination of UDE and its fragments using VUVCD and SELCON3 program. **(A)** Vacuum-ultraviolet circular dichroism (Δε) spectra of the UDE protein and its nine truncated fragments measured over the wavelength region of λ = 170–255 nm. The spectra are sorted into two panels for better visibility and spectra of UDE (red) is shown in both panels for reference **(B)** Decomposition of the CD spectra of UDE and its selected fragments using six secondary structure components; regular/distorted α-helix (rH/dH), regular/distorted β-strand (rS/dS), turn (T), and disordered structure (D). Upper panel: CD spectrum of UDE as measured and as fitted using the six components [22–24]. Spectra of the components are also plotted with magnitudes proportional to their ratios in the full-length protein. Lower Panel: Difference spectra corresponding to UDE-NA2 and NG3-NA3 together with the fittings based on the spectra of the six basic components.

The spectral shape for UDE, shown in Fig 4A, indicates high α-helix content. Similar to the CD spectra of regular α-helices, the spectrum of UDE is characterized by two negative peaks at 222 and 208 nm, and a positive peak at 192 nm [36]. The minimum at 222 nm is due to the nπ* transition of the carbonyl group of the peptide bond, while the parallel and perpendicular excitation of the peptide ππ* transition is responsible for the other minimum at 208 nm and the positive peak at 192 nm [8,36]. An additional negative band at around 170 nm and a positive shoulder at 175 nm were observed; these are characteristic of α-helical structures in former VUVCD measurements [34,35]. While the spectral shape resembles of the delta epsilon spectrum of α-helical structures, its overall magnitude is about a factor of two smaller, implying that a considerable portion of the sequence forms secondary structures different from α-helices or remains disordered.

**Fig 5:**
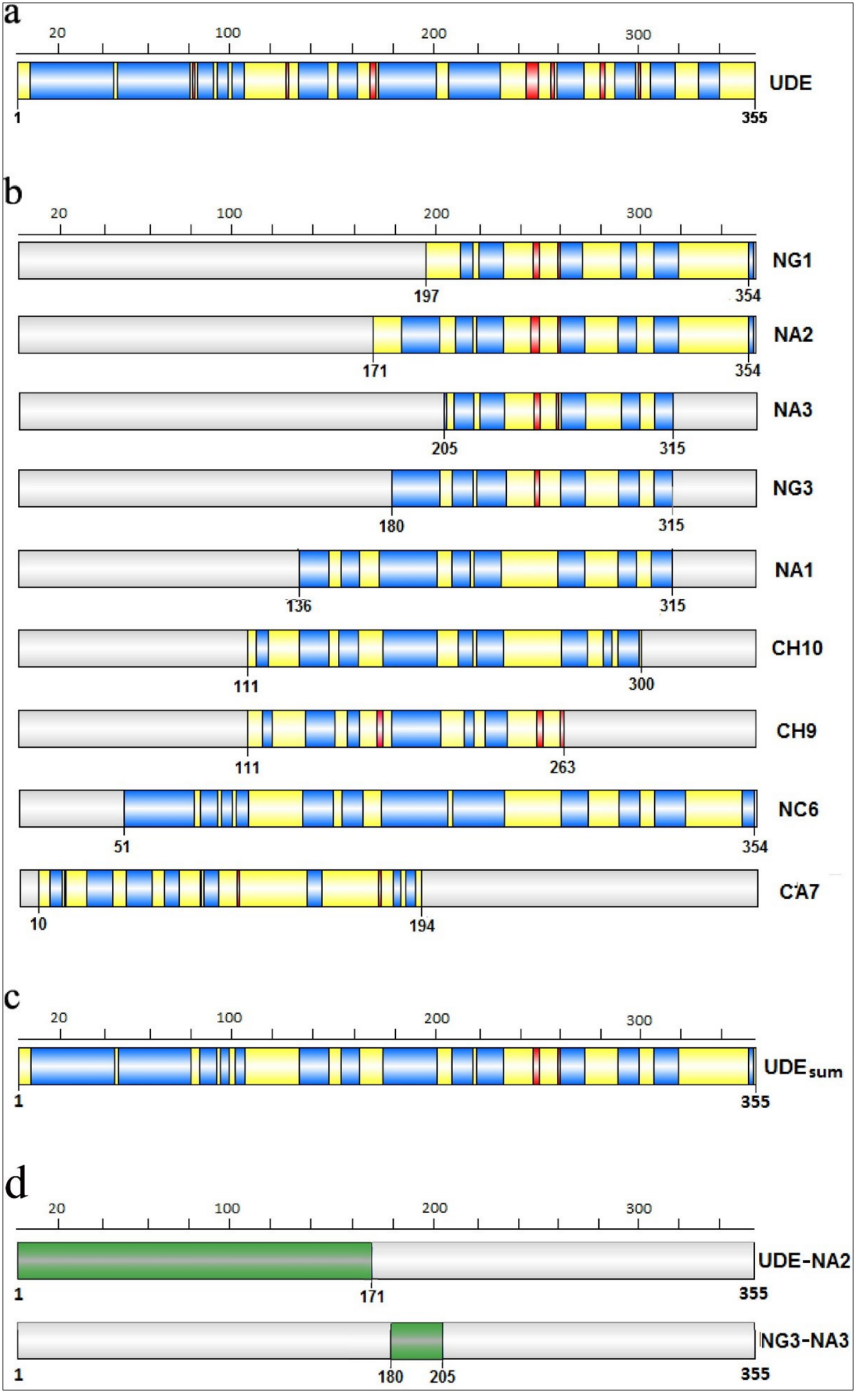
Spatial distribution of the secondary structure components along UDE protein. The location of α-helical segments **(A)** in the full-length (355 aa) UDE protein and **(B)** in its nine truncated fragments were determined from the CD spectra and the amino acid sequence using a neural network algorithm [25,26]. The α-helical segments and β-strands are displayed in blue and red, respectively, while both turns and disordered parts appear in yellow. **(C)** Our final estimate for the secondary structure of UDE obtained as an average of the structure of UDE proposed in panel **(A)** and the structure of the fragments shown in panel **(B)** except for CA7. **(D)** The native structure of the N-terminal end of the full-length UDE was investigated using the evaluation of the CD spectrum of the N- terminal as the difference between the CD spectra of UDE and NA2 according to (Δε1UDE×N1UDE−Δε1NA2×N1NA2)/(N1UDE—N1NA2), where Δε is the molar ellipticity and N is the number of amino acids for UDE and NA2. The same subtraction method was performed with the highly overlapping fragments NG3 and NA3.

Quantitative evaluation of the spectral data was performed by the SELCON3 program with the VUVCD spectra of 31 reference proteins over the wavelength region of 170–255 nm [28,29]. Based on the experimental data, we estimated the relative ratios of the secondary structure elements: 62% α-helix (40% regular and 22% distorted helix), 8% β-strand (2% regular and 6% distorted strand), 9% turn and 21% disordered component as shown in Fig 4B. From the portion of the distorted parts located at the edges of the regular regions, we estimate the number of α-helical segments in UDE to be n_α_ = 20 [27,30]. On the other hand, the anomalously high portion of distorted parts in β-strands may imply that the corresponding structures cannot be unambiguously classified as β-strands and are hardly distinguishable from disordered structures. We performed a neural network analysis treating the amino acid sequence and the secondary structure contents (obtained from the CD spectrum) with equal weighting to determine the location of the α-helical and β-strand segments, i.e. to obtain the spatially resolved secondary structure of UDE [31]. The resulting structure, displayed schematically in Fig 5A and 5B, shows a good correlation between the CD spectrum and the amino acid sequence, since the portion of each secondary-structure component has been affected by less than 2–3%. Though the presence of short β-strand regions is supported by the neural network algorithm, we found that this method strongly underestimates the number of α-helical and β-strand segments in comparison with the results solely based on the CD spectrum of the protein. Such tendency to merge neighbouring α-helical or β-strand segments separated by a few amino acids with disordered structure have already been reported in the literature [31]. The CD spectra of the nine fragments of UDE indicate that the exclusive α-helical content is preserved in the fragments, especially in case of NA1, NC6, NG3 and NA3 (Fig 4A). Indeed the results of the neural network analysis, shown in Fig 5A and 5B, verify that the native structure is conserved in the inner regions of the fragments, except for CA7 where the truncation process is followed by a clear transformation of the structure. By averaging the results obtained for the fragments with those of the full-length protein in the overlapping regions, we could further improve the reliability of the secondary structure estimation for UDE. Our final estimate for the secondary structure of UDE is given in Fig 5C. From the averaging process the edge regions of the fragments (five amino acids) and the full CA7 were excluded, since they do not preserve their native structure.

Exploiting the stable structural characteristic of most of the UDE fragments, we demonstrate that the secondary structure can be spatially resolved along the sequence solely based on the CD spectra. As an example, we investigate the native structure of the N-terminal end of the UDE protein (see Fig 5D), since the neural network analysis predicted significant differences between this part in the intact protein and the corresponding CA7 fragment. We estimate the CD spectrum of the N-terminal end as the difference between the CD spectra of UDE and NA2 according to (Δε_1UDE_×N_1UDE_−Δε_1NA2_×N_1NA2_)/(N_1UDE_—N_1NA2_), where Δε is the molar ellipticity and N is the number of amino acids for UDE and NA2. This difference spectrum, displayed in Fig 4B, clearly shows that the N-terminal end is purely α-helical (74% regular helix, 18% distorted helix, 4% regular strand and 4% disordered) when embedded in the full-length protein. The efficiency of this subtraction method is more obvious when comparing highly overlapping fragments such as NG3 and NA3 as indicated in Fig 5D. The difference spectrum corresponding to a 25 aa part of the sequence again shows exclusive α-helix content (65% regular helix, 15% distorted helix and 20% turn). Besides the high quality CD spectral information, we emphasize that the conservation of the native structure is required for the fragments used in such comparisons.

## Discussion

In this study we continued the structural characterization of the unique uracil-DNA degrading factor utilizing the preliminary domain organization analysis and secondary structure prediction for the N-terminal end of UDE. Secondary structure prediction suggested that the duplicated fragments (Motifs 1A-1B) are mainly α-helical and interact through a conserved surface segment. Structural disorder predictors indicated that the UDE protein possesses flexible segments at both the N- and C-termini, and also in the linker regions of the conserved motifs. However, the secondary structural content of the well-folded C-terminal domain (containing Motifs 2–4) was still not clear [15]. The ESPRIT library-based screening approach [2,18] was applied to UDE yielding well-folded, highly expressing, soluble truncated UDE fragments that had not been possible to design prior to this approach. We discerned nine clusters of expressing constructs and the foldedness, thermostability and oligomeric status of one representative of each cluster were examined. The analysis indicated that CH9, CH10 and CA7 variants, though well-expressed, contain disordered or flexible residues that potentially inhibit well-defined tertiary/quaternery structure formation. Using multiple sequence alignments we found that five clusters determine potential new domain boundaries compared to the distribution of conserved motifs in UDE homologs from pupating insects. The CH9 and CH10 constructs comprising the previously unpredicted domain termination with different terminal residue would inhibit the chances of crystallization and functional characterization based on the result of biophysical investigation. We performed detailed analyses of UDE deletions on secondary structure content by processing the data from VUVCD spectroscopy [22,34,35]. Quantitative evaluation of VUVCD data by SELCON 3 [27–30] and a neural network algorithm [31] determined the number and location of α-helical segments in both the fragments and the full-length UDE. Data confirmed the mainly α-helical content for the full-length protein, which is preserved in the truncated constructs, also for the compact C-terminal end of UDE. Comparison of the secondary structure content of the N-terminal duplicated domains showed that the previously determined boundaries of conserved motifs do not correspond exactly to the predicted arrangement of α-helical segments. α-helical structures are common in DNA binding motifs such as helix-turn-helix [37], leucine zipper/coiled-coil [38] and zinc finger motifs [39]. Previous results showed that both full length UDE and a truncated naturally occurring isoform bind normal and uracil-containing DNA, but degrade only uracil containing DNA [13,15]. We suspect that the helix-turn-helix nucleic acid binding structural motif may be present in full length UDE and in the truncated constructs studied here, which is formed either by the positively charged N-terminal segments or the α-helical C-terminal domain. Furthermore, the oligomerization of the truncated constructs may represent the tertiary helix-helix interactions also in the full-length protein. We showed that a combination ESPRIT to produce well-behaving local regions of a target protein and VUVCD spectroscopy to determine secondary structure therein, can provide clear new insights into its structural composition. We performed our analysis using UDE, an interesting protein containing both unstructured segments and independently folded domains. A major advantage of VUVCD spectroscopy is that it may be applicable to dilute solutions of proteins with large molar mass, where X-ray and nuclear magnetic resonance techniques cannot be applied.

## Supporting Information

S1 Fig Expression and purification of UDE segments followed by SDS-PAGE analysis of samples at the different extraction and chromatography steps. SDS-PAGE gels are shown for all the UDE segments characterized in details our study. Abbreviations are as follows: Sup: supernatant after cell lysis; Flow: flow-through from the Ni-NTA column; Res: wash with buffer A; Salt: wash with buffer B; 25, 50, or 300 mM: elution with buffer A also containing 25, 50 or 300 mM imidazole, respectively; El.1–3 or 1–5, elution fractions 1–3 or 1–5; MM: molecular marker. Lanes labelled with Sup. Flow., Res, and Salt contain samples from the supernatant of cell lysis, the flowthrough on the Ni-column, the wash of the Ni-column with buffer A, and the wash of the Ni-column with buffer B (buffer A complemented with 1 M NaCl). UDE constructs were eluted with buffer A complemented with the imidazole concentrations indicated on the gel lanes. MM: molecular marker.
(PDF)
