# Supplementary material for: Secondary Structure Prediction of Protein Constructs Using Random Incremental Truncation and Vacuum-Ultraviolet CD Spectroscopy
Source: PLoS One. 2016 Jun 7;11(6):e0156238. doi: 10.1371/journal.pone.0156238 (PMC4896422; doi:10.1371/journal.pone.0156238)
Supplement: S1 Fig — SDS-PAGE gels are shown for all the UDE segments characterized in details our study. Abbreviations are as follows: Sup: supernatant after cell lysis; Flow: flow-through from the Ni-NTA column; Res: wash with buffer A; Salt: wash with buffer B; 25, 50, or 300 mM: elution with buffer A also containing 25, 50 or 300 mM imidazole, respectively; El.1–3 or 1–5, elution fractions 1–3 or 1–5; MM: molecular marker. Lanes labelled with Sup. Flow., Res, and Salt contain samples from the supernatant of cell lysis, the flowthrough on the Ni-column, the wash of the Ni-column with buffer A, and the wash of the Ni-column with buffer B (buffer A complemented with 1 M NaCl). UDE constructs were eluted with buffer A complemented with the imidazole concentrations indicated on the gel lanes. MM: molecular marker. (PDF) [file pone.0156238.s001.pdf]

Supplementary Information for the study:

Secondary structure prediction of protein constructs using random incremental truncation and vacuum-ultraviolet CD spectroscopy

Mária Pukáncsik[1,2*], Ágnes Orbán[2], Kinga Nagy[1], Koichi Matsuo[3], Kunihiko Gekko[3], Damien Maurin[4], Darren Hart[4], István Kézsmárki[2] and Beáta G. Vértessy [1,5*]

[1] Institute of Enzymology, Research Centre for Natural Sciences, Hungarian Academy of Sciences, Budapest, Hungary, [2] Department of Physics, Budapest University of Technology and Economics and MTA-BME Lendület Magneto-optical Spectroscopy Research Group,

1111 Budapest, Hungary, [3] Hiroshima Synchrotron Radiation Center, Hiroshima University, Higashi-Hiroshima, Japan, [4] Institut de Biologie Structurale (IBS), CEA, CNRS, University Grenoble Alpes, Grenoble 38044, France, [5] Department of Applied Biotechnology, Budapest University of Technology and Economics, Budapest, Hungary

*Corresponding author : vertessy@mail.bme.hu; vertessy.beata@ttk.mta.hu; p_marcsi@enzim.hu

Supplementary Information contains:

S1 Figure Expression and purification of UDE segments followed by SDS-PAGE analysis of samples at the different extraction and chromatography steps. SDS-PAGE gels are shown for all the UDE segments characterized in details in our study. Abbreviations are as follows: Sup: supernatant after cell lysis; Flow: flow-through from the Ni-NTA column; Res: wash with buffer A; Salt: wash with buffer B; 25, 50, or 300 mM: elution with buffer A also containing 25, 50 or 300 mM imidazole, respectively; El.1-3 or 1-5, elution fractions 1-3 or 1-5; MM: molecular marker.

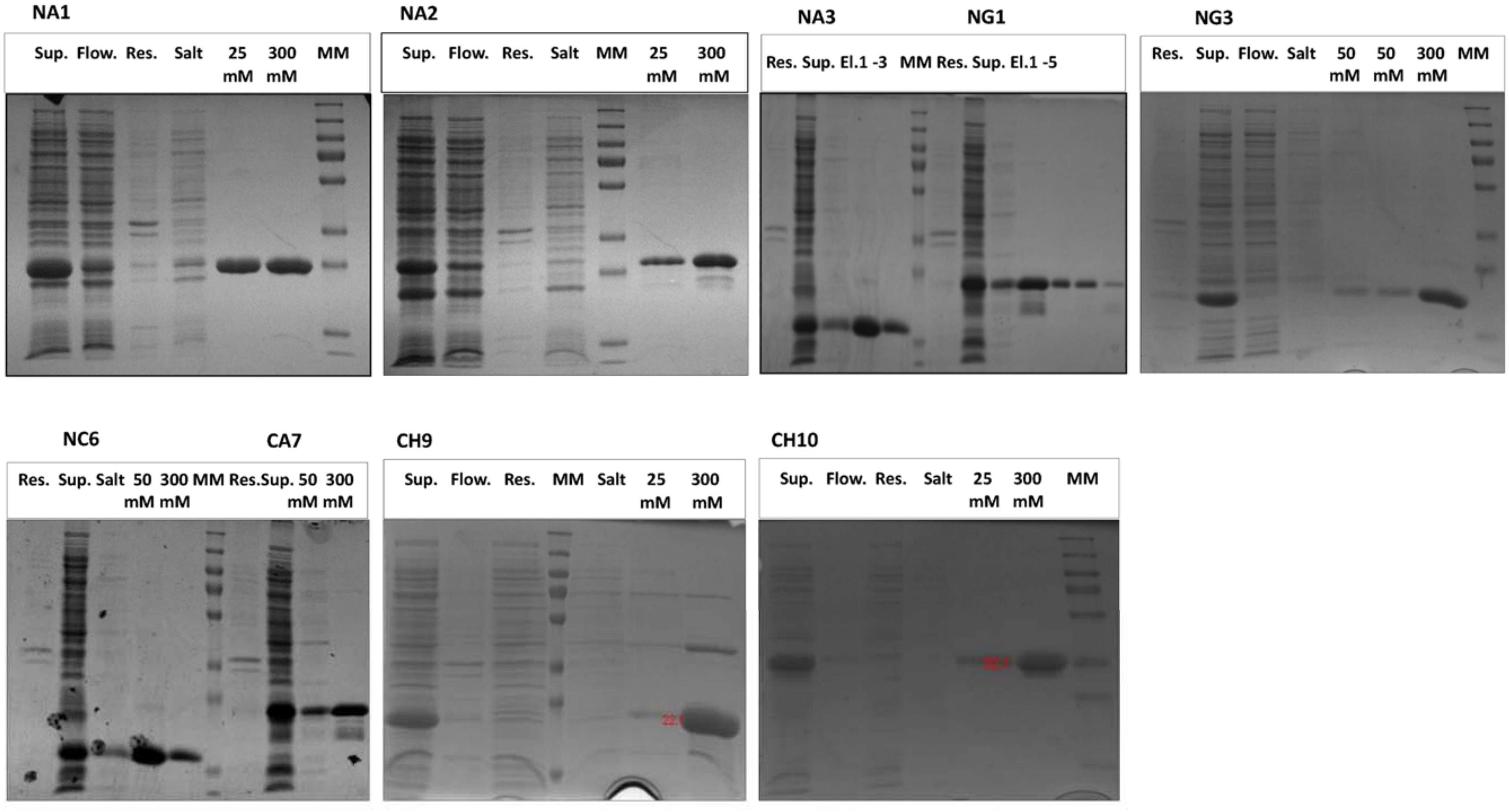

S1 Figure **S1 Figure Expression and purification of UDE segments followed by SDS-PAGE analysis of samples at the different extraction and chromatography steps.** SDS-PAGE gels are shown for all the UDE segments characterized in details in our study. Abbreviations are as follows: Sup: supernatant after cell lysis; Flow: flow-through from the Ni-NTA column; Res: wash with buffer A; Salt: wash with buffer B; 25, 50, or 300 mM: elution with buffer A also containing 25, 50 or 300 mM imidazole, respectively; El.1-3 or 1-5, elution fractions 1-3 or 1-5; MM: molecular marker.
